# Liver involvement by multiple myeloma presenting as hypervascular focal lesions in a patient with chronic hepatitis B infection

**DOI:** 10.1259/bjrcr.20150013

**Published:** 2016-05-08

**Authors:** Magda Marcon, Lorenzo Cereser, Rossano Girometti, Palmina Cataldi, Stefano Volpetti, Massimo Bazzocchi

**Affiliations:** ^1^Department of Medical and Biological Sciences, Institute of Diagnostic Radiology, University of Udine, University Hospital “Santa Maria della Misericordia”, Udine, Italy; ^2^Department of Medical and Morphological Sciences, University of Udine, University Hospital “Santa Maria della Misericordia”, Udine, Italy; ^3^Department of Experimental and Clinical Sciences, Division of Hematology and Cellular Therapies Unit “Carlo Melzi”, University Hospital “Santa Maria della Misericordia”, Udine, Italy

## Abstract

Extramedullary myeloma refers to the infiltration of neoplastic monoclonal plasma cells in either organs or soft tissues. The disease is clinically and radiologically underestimated compared with the autopsy findings and is usually associated with a more aggressive clinical course and poorer outcome. A minority of patients with extramedullary myeloma show hepatic involvement, usually in the form of diffuse parenchymal infiltration. When focal infiltration is present, variable imaging findings have been described both on CT scan and MRI. We report the case of a 63-year-old male with hepatitis B virus-related liver disease and biopsy-proven multiple myeloma involving the liver, manifesting as hypervascular focal liver lesions on MRI. A brief review of the literature is also proposed.

## Summary

Extramedullary multiple myeloma (e-MM) is a rare but clinically and radiologically underestimated entity that is associated with poor prognosis.^1^

An increasing incidence of e-MM has been reported in recent decades, both at diagnosis and during follow-up. The latter may be partly explained by the widespread use of novel therapeutic agents that have led to a significant improvement in survival. In addition, the use of more sensitive imaging techniques (*i.e.* CT scan, MRI and 18–fludeoxyglucose-positron emission tomography), recently included into the routine staging systems, such as the Durie–Salmon Plus, may also have contributed to the increasing detection of e-MM lesions.^2^ Younger patients or patients relapsing after stem cell transplantation are the ones most commonly affected.

Although hepatic involvement is observed at autopsy in up to 30% of patients, ante-mortem diagnosis is significantly less common. Diffuse and nodular patterns of plasma cell infiltration have been reported, the former being more common.^3^ Imaging appearances of hepatic multiple myeloma (MM) manifesting with focal/multifocal pattern are non-specific, and heterogeneous features have been described in few published reports and case series so far.^3–11^ Most often, focal liver lesions have been observed as non- or mildly enhancing on CT scan and as moderate or minimally enhancing on MRI; hepatic involvement has been noted in only one case, described as hypervascular liver lesions on CT scan (Table 1).^11^ Nevertheless, a proper comparison among the different patterns is not feasible as most of the studies did not describe the details of the CT scan and MR techniques applied. The differential diagnosis of hypervascular focal liver lesions mainly includes hepatocellular carcinoma and hypervascular metastases, while hepatic involvement by MM is not routinely considered.^12,13^

**Table 1.** Imaging features of myelomatous nodular involvement of the liver

Publication	Ultrasound	CT	MRI
Mathieu et al^5^		Hypodense, enhancing from the periphery to the centre	
Nguyen et al^6^	“Target” appearance	Hypodense	*T*_1_ weighted: hypointense outer layer, hyperintense rim, isointense core *T*_2_ weighted: hyperintense outer layer, hypointense rim, hyperintense core
Kelekis et al^7^	“Target” appearance	Enlarged liver without discrete nodules in unenhanced phase	*T*_1_ and *T*_2_ weighted: hyperintense Moderate enhancement
Ng et al^8^	“Target” appearance	Slightly hypodense, mild enhancement	*T*_1_ weighted: slightly hypointense *T*_2_ weighted: hyperintense Minimal enhancement
Patlas et al^9^	Hypoechoic/mixed echogenicity	Hypodense on unenhanced and post-contrast phases	
Monill et al^10^		Non-enhancing nodules	
Tan et al^11^		Arterial enhancement Isodense/mildly hypodense on delayed phase	
Present case	Hypoechoic	Arterial enhancement Isodense on portal venous and delayed phase	*T*_1_ weighted: slightly hypointense *T*_2_ weighted: hyperintense Arterial enhancement with contrast washout and capsule appearance on portal venous phase Diffusion-weighted imaging: restricted diffusion

“Target” appearance: hypoechoic halo surrounding the echogenic/isoechoic core.

We present the ultrasound, CT scan and MRI features, and the corresponding pathological findings of hepatic myelomatous involvement manifesting as hypervascular liver lesions in a patient with MM and hepatitis B virus (HBV)-related liver disease.

## Clinical presentation

A 63-year-old male with a diagnosis of HBV infection [hepatitis B surface antigen (HBsAg) positivity, HBV DNA 2578 IU ml^−1^], on treatment with tenofovir, was admitted to our hospital for routine abdominal ultrasound examination. The patient had immunoglobulin Gκ MM that was diagnosed in 2001 and staged as III A according to the Durie–Salmon staging system;^2^ he had been heavily pretreated and had relapsed multiple times. Previous treatments included four courses of vincristine, adriamycin and dexamethasone; thalidomide; two autologous stem cell transplantation procedures; four cycles of bortezomib and dexamethasone; multiple bisphosphonate infusions; several radiotherapy treatments for bone lesions; and finally allogeneic stem cell transplantation from a matched, unrelated donor performed in December 2011. At the time of the ultrasound examination, the patient was receiving donor lymphocyte infusions owing to further relapse, including vertebral and sacral bone lesions and increased serum monoclonal protein levels; laboratory hepatic damage markers, in particular aspartate aminotransferase and alanine aminotransferase, were only slightly increased, while bilirubin was normal. Alpha fetoprotein level was also measured and was normal (6.3 ng ml^−1^, normal range < 8 ng ml^−1^).

## Investigations/imaging findings

**Figure 1.**
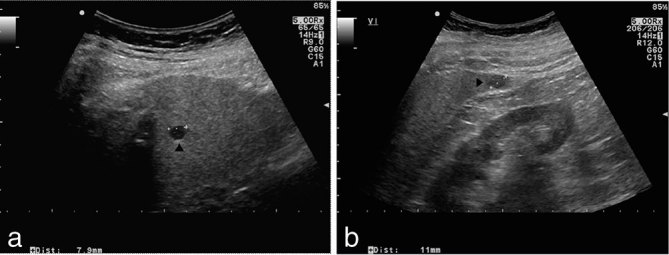
Ultrasound imaging of the liver showing a hypoechoic, well-delimited lesion in segment 8 (a, black arrowhead) and 6 (b, black arrowhead) measuring 8 and 11 mm in maximum diameter, respectively. Mild diffuse increased echogenicity of the liver parenchyma is also present, consistent with fatty infiltration.

**Figure 2.**
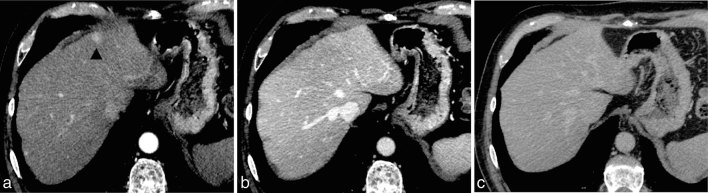
(a) A contrast-enhanced axial CT image acquired during the hepatic arterial phase demonstrates an enhancing lesion measuring approximately 14 mm in the left lobe of the liver (black arrowhead), which shows isodensity to the surrounding parenchyma in the images acquired during the portal venous phase (b) and the delayed phase (c).

**Figure 3.**
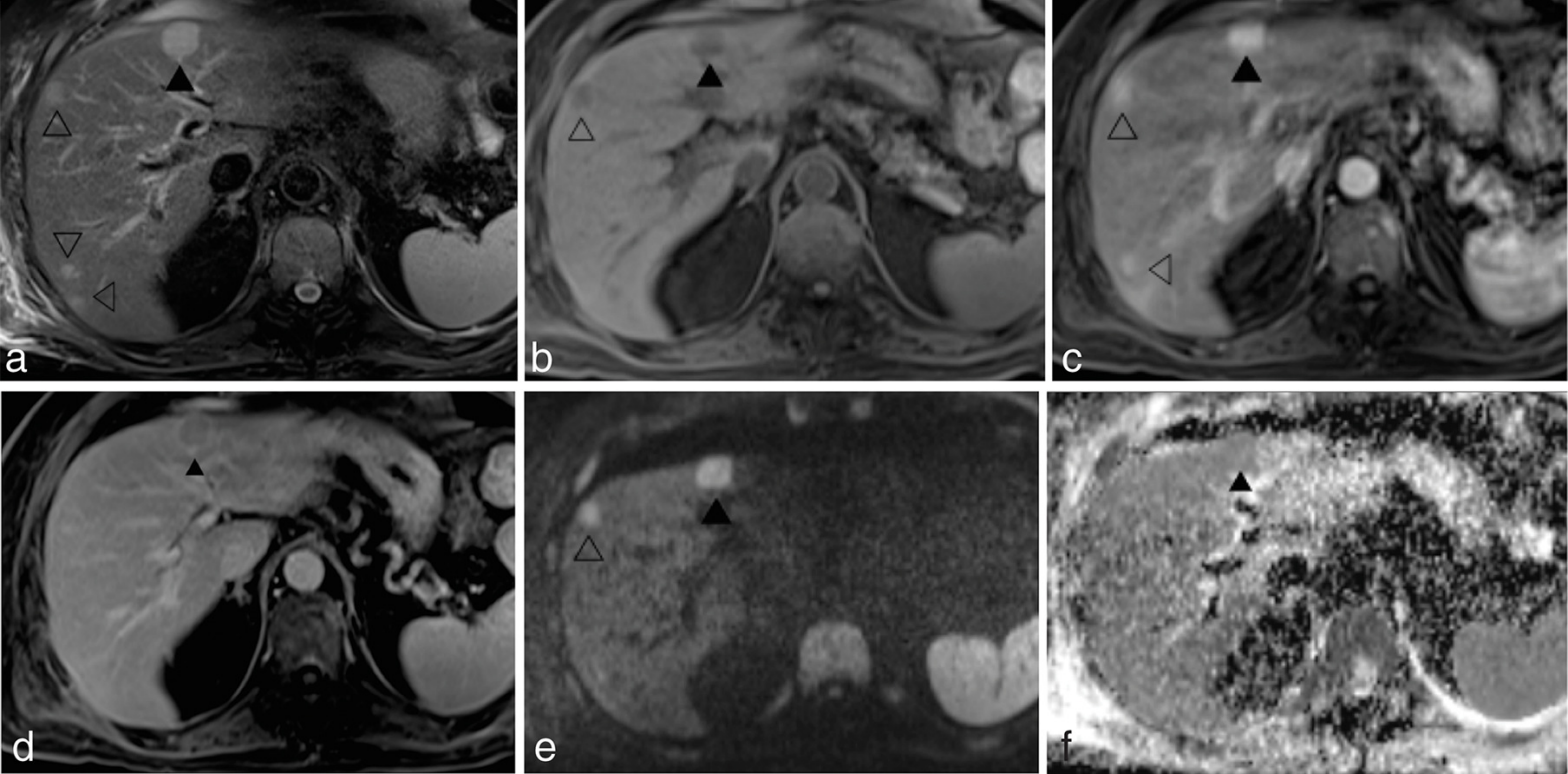
MR images acquired 1 month after the CT scan at the same level as those in Figure 2. The lesion on the left lobe (black arrowheads) measures 21 mm and shows hyperintense signal on turbo spin echo-spectral adiabatic inversion recovery *T*_2_ weighted image (a) and hypointense signal on fat-saturated volumetric interpolated breath-hold examination *T*_1_ weighted image (b). The corresponding gadobenate dimeglumine-enhanced fat-saturated volumetric interpolated breath-hold examination *T*_1_ weighted images show intense lesion enhancement during the hepatic arterial phase (c) and the presence of contrast washout and capsule appearance during the delayed phase (d). On diffusion-weighted imaging (*b* = 800 s mm^−2^) (e), the lesion demonstrates high signal intensity but without evidence of restricted diffusion on the apparent diffusion coefficient map (f). Compared with the CT scan acquired at the same level, additional lesions (empty arrowheads) are visible in the right lobe of the liver; all of them show intense enhancement during the hepatic arterial phase but without contrast washout during the portal and delayed phases. The largest lesion in the left liver lobe (black arrowhead) was biopsied.

**Figure 4.**
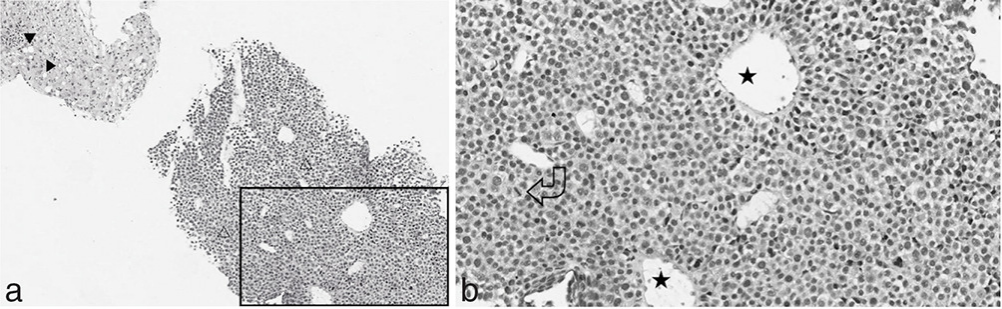
Photomicrographs of the histopatological specimen showing (a) diffuse infiltration of the liver by monomorphic plasmacytoid cells with hyperchromatic nuclei (empty arrowheads). The adjacent hepatic parenchyma shows macro- and microvesicular steatosis (black arrowheads) (hematoxylin and eosin ×4). (b) The selected area in (a) is shown with greater magnification, better demonstrating atypical plasma cells and mitotic activity (curved arrow), and the presence of neoformed vascular structure (stars) (×10).

**Figure 5.**
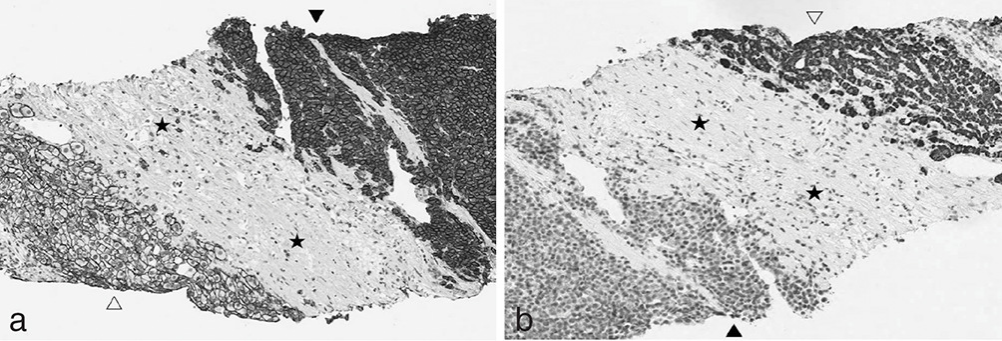
(a) Immunohistochemical staining shows CD-138 cytoplasmatic and plasma membrane expression in plasmacytoid cells (black arrowhead), whereas hepatocytes (empty arrowhead) and fibroblasts (stars) show only plasma membrane positivity and complete absence of CD-138 expression, respectively. (b) Immunohistochemical analysis of hepatocyte-specific antigen antibody (OCH1E5) expression shows cytoplasmatic staining in hepatocytes (empty arrowhead) and lack of significant expression in plasmacytoid cells (black arrowhead) and fibroblasts (stars) (×10).

Ultrasound imaging showed five well-circumscribed hypoechoic focal lesions measuring 5–12 mm in maximum diameter, distributed throughout the liver (Figure 1). A mild diffuse increased echogenicity of the liver was also present, consistent with fatty infiltration, without clear ultrasound signs of cirrhosis. A multiphasic contrast-enhanced CT scan of the abdomen was performed to better characterize the lesions and revealed the presence of nine focal liver nodules measuring 6–16 mm in maximum diameter. The lesions were isodense on pre-contrast CT scan, showing mild contrast enhancement on hepatic arterial phase images and isoenhancing to the surrounding liver on portal venous and delayed phase images (Figure 2). MRI examination was subsequently performed (1 month later). In our institution, the MRI protocol (Table 2) for the upper abdomen includes multiple breath-hold gradient echo in-phase and out-of-phase *T*_1_ weighted imaging, a respiration-triggered spectral adiabatic inversion recovery turbo spin echo *T*_2_ weighted sequence and a dynamic study using volumetric interpolated breath-hold examination *T*_1_ weighted sequence with fat saturation before and after i.v. injection of 0.1 ml kg^−1^ of gadobenate dimeglumine (MultiHance, Bracco, Milan, Italy). The latter sequence was repeated on the axial plane 30, 70 and 300 s after contrast administration and in the hepato-specific phase at about 1 h after contrast administration. Diffusion-weighted imaging was performed before the dynamic study with a respiration-triggered single-shot echo-planar sequence acquired on the axial plane with *b* values of 50, 400 and 800 s mm^−2^. An apparent diffusion coefficient map was obtained. Multiple focal liver lesions measuring 10–25 mm in maximum diameter were depicted, appearing hyperintense on *T*_2_ weighted images and hypointense on pre-contrast *T*_1_ weighted images. All the lesions showed hyperenhancement on hepatic arterial phase images; the largest ones (three lesions > 20 mm) showed hypoenhancement to the surrounding liver parenchyma (*i.e.* washout appearance) and capsule appearance on the portal venous and delayed phases (Figure 3), whereas others exhibited isointense signal to the surrounding liver during both the portal venous and delayed phases. Moreover, all the lesions were hypointense on the hepatobiliary phase images. All the lesions were hyperintense on diffusion-weighted images (*b* = 800 s mm^−2^) but showed variable restriction on the apparent diffusion coefficient map. No signal dropout was appreciable on the *T*_1_ weighted gradient echo out-of-phase images compared with the in-phase images to suggest intralesional fat. No additional pathological findings were detected. Percutaneous ultrasound-guided liver biopsy of the largest subcapsular lesion on the left lobe (Figure 3) was performed using an 18-gauge cutting needle and histopatological and immunohistochemical analysis revealed the presence of plasma cells with anaplastic features, consistent with liver involvement by MM. The presence of neoformed vascular structures was also demonstrated, in line with the hypervascularity observed on CT scan and MRI (Figures 4 and 5).

**Table 2.** MRI protocol. Diffusion-weighted imaging was performed by using three *b* values (50, 400 and 800 s mm^−2^) within the same acquisition

Sequence parameters	2D GRE	2D TSE SPAIR	3D GRE VIBE with fat-saturation	EPI
Weighting	*T*_1_	*T*_2_	*T*_1_	Diffusion
Orientation	Transversal	Transversal	Transversal	Transversal
Repetition time (ms)	118	1700	4.23	1900
Echo time (ms)	2.35/5.04	65	1.48	69
Field of view (mm)	337*400	285*380	275*400	285*380
Matrix size	230*256	259*320	179*256	153*192
Section thickness (mm)	6	6	4	6
Intersection gap (%)	20	30	20	30
Number of sections	27	25	60	25
Number of signals acquired	1	1	1	2
Acquisition time (s)	24	110	12	187

2D, two-dimensional; 3D, three-dimensional; EPI, echo-planar imaging; GRE, gradient echo; SPAIR, spectral adiabatic inversion recovery; TSE, turbo spin echo; VIBE, volumetric interpolated breath-hold examination.

## Differential diagnosis

Differential diagnoses of the focal liver lesions in our case included the following:

- hepatocellular carcinoma
- hypervascular metastatic disease (*i.e.* melanoma, primary neuroendocrine tumours, renal cell carcinoma, thyroid carcinoma and sarcoma)
- hepatic MM.

## Treatment

The patient was treated with four cycles of vincristine, cyclophosphamide and doxorubicin and underwent close clinical and laboratory monitoring.

3 months after starting this treatment, an abdominal ultrasound examination was performed: the previously described hepatic lesions were not apparent anymore and no new lesions were observed. The overall clinical and laboratory status was slightly improved as well.

## Outcome and follow-up

To the best of our knowledge, data concerning the prognostic impact of hepatic e-MM are not available but, more generally, the presence of e-MM has been associated with an aggressive course. Indeed, the presence of e-MM at any time during the course of the disease is associated with shorter progression-free survival and overall survival.^1^

Periodic evaluation for progression of MM is recommended, including a complete history and physical examination as well as laboratory tests.

## Learning points

1. In patients with MM, care should be taken about considering extramedullary myelomatous localization in the differential diagnosis of hypervascular lesions of the liver.
2. Imaging appearances of hepatic MM manifesting with focal/multifocal pattern are heterogeneous.

## Consent

Informed consent to publish this case, including images and data, was obtained and is held on record.
